# A198 RELATIONSHIP BETWEEN HEPATIC GENE EXPRESSION AND MICROBIOME ACCORDING TO DISEASE SEVERITY IN OBESE PATIENTS WITH NON-ALCOHOLIC FATTY LIVER DISEASE

**DOI:** 10.1093/jcag/gwad061.198

**Published:** 2024-02-14

**Authors:** Y Ghorbani, K Schwenger, A Teterina, Y Liu, W Lou, S Fischer, T Jackson, A Okrainec, J P Allard

**Affiliations:** University Health Network, Toronto, ON, Canada; University Health Network, Toronto, ON, Canada; University of Toronto, Toronto, ON, Canada; University of Toronto, Toronto, ON, Canada; University of Toronto, Toronto, ON, Canada; University Health Network, Toronto, ON, Canada; University Health Network, Toronto, ON, Canada; University Health Network, Toronto, ON, Canada; University Health Network, Toronto, ON, Canada

## Abstract

**Background:**

Non-alcoholic fatty liver disease (NAFLD) ranges from steatosis to inflammation (steatohepatitis; NASH), fibrosis and cirrhosis. Development and progression of NAFLD/NASH is attributed to factors such as obesity and metabolic syndrome as well as intestinal microbiome (IM) but research is limited on the relationship between IM and hepatic gene expression.

**Aims:**

Our objective was to assess in obese patients;1) the transcriptome signature of NAFLD and fibrosis and; 2) determine the relationship between the identified genes and IM.

**Methods:**

Biochemical and anthropometric measurements as well fecal samples for IM shotgun metagenomic sequencing were collected in those with obesity undergoing bariatric surgery. Liver histology was assessed using Brunt scoring system and hepatic transcriptome was assessed using RNA-Seq. Differences in biochemical data and IM were determined using Kruskal-Wallis test followed by Wilcoxon ranked sum test. Differentially expressed genes (DEGs) in the liver were determined using DESeq2 package in R and had |log2 foldchange| ≥1 with adjusted p-value ampersand:003C0.05. Spearman correlation coefficients was used to evaluate the association between significant DEGs and significant bacteria and adjusted using Benjamini-Hochberg.

**Results:**

93 patients had both transcriptome and IM data. Of those, 27 had normal liver obese (NLO) and 66 had NAFLD (including 31 NASH). In those with NAFLD, 19 had no fibrosis (F0) and 47 had presence of fibrosis (F1-F4). We found 71 significantly different DEGs (e.g. PPP1R3G ) in NASH vs NLO and 12 (e.g. THBS2 and GLI2) in NAFLD with presence of fibrosis vs no fibrosis. Those with NASH generally had higher *Escherichia coli*, *Blautia hansenii* and lower *Alistipees putredinis*. Those with NAFLD and fibrosis had increased *E. coli* and reduced *Eubacterium ventriosum* and *A. putredinis*. In the preliminary analysis of NASH vs NLO, *B. hansenii* negatively correlated with glucose homeostasis-related PPP1R3G. In those with NAFLD, *A. putredinis* correlated negatively with THBS2 (i.e. Thrombospondin-2) and, in patients with NAFLD and fibrosis, *E. coli* correlated positively with GLI2 (related to activation of hepatic stellate cells).

**Conclusions:**

DEGs were associated with specific bacteria in NASH or fibrosis. Next steps include analyzing the microbial metagenomic pathways as well as metabolites and determine the relationship between the IM, hepatic transcriptome and metabolites according to disease severity.

**Funding Agencies:**

CIHRAmerican College of Gastroenterology Bridge Award, BBDC Tamarack Graduate Award in Diabetes Research

